# Investigation of factors regarding the effects of COVID-19 pandemic on college students’ depression by quantum annealer

**DOI:** 10.1038/s41598-024-54533-8

**Published:** 2024-02-26

**Authors:** Junggu Choi, Kion Kim, Soo Hyun Park, Juyoen Hur, Hyunjung Yang, Young-Hoon Kim, Hakbae Lee, Sanghoon Han

**Affiliations:** 1 Yonsei Graduate program in Cognitive Science, Yonsei University, Seoul, 03722 Republic of Korea; 2 Korea Quantum Computing©, Seoul, 06164 Republic of Korea; 3 Department of Psychology, Yonsei University, Seoul, 03722 Republic of Korea; 4 University College, Yonsei University, Seoul, 03722 Republic of Korea; 5 Deparment of Applied Statistics, Yonsei University, Seoul, 03722 Republic of Korea; 6 Department of Statistics and Data Science, Yonsei University, Seoul, 03722 Republic of Korea

**Keywords:** Environmental social sciences, Health care, Medical research, Risk factors, Engineering

## Abstract

Diverse cases regarding the impact, with its related factors, of the COVID-19 pandemic on mental health have been reported in previous studies. In this study, multivariable datasets were collected from 751 college students who could be easily affected by pandemics based on the complex relationships between various mental health factors. We utilized quantum annealing (QA)-based feature selection algorithms that were executed by commercial D-Wave quantum computers to determine the changes in the relative importance of the associated factors before and after the pandemic. Multivariable linear regression (MLR) and XGBoost models were also applied to validate the QA-based algorithms. Based on the experimental results, we confirm that QA-based algorithms have comparable capabilities in factor analysis research to the MLR models that have been widely used in previous studies. Furthermore, the performance of the QA-based algorithms was validated through the important factor results from the algorithms. Pandemic-related factors (e.g., confidence in the social system) and psychological factors (e.g. decision-making in uncertain situations) were more important in post-pandemic conditions. Although the results should be validated using other mental health variables or national datasets, this study will serve as a reference for researchers regarding the use of the quantum annealing approach in factor analysis with validation through real-world survey dataset analysis.

## Introduction

The coronavirus disease 2019 (COVID-19) pandemic has significantly disrupted global society in diverse aspects^1,2^. Researchers have attempted to investigate the influence of pandemics on various domains, including biological or psychological areas in previous studies^3–5^. Among the several domains impacted by the pandemic, mental health issues during the pandemic received attention as they were associated with regulations, including social distancing^6,7^. In particular, researchers have focused on college students, who can be influenced more easily than other age groups, to investigate differences or variations in their mental health status during the pandemic. For example, several researchers have emphasized the importance of long-term monitoring and mental health support for college students, with experimental results of the analyses of the determinants of mental health^8,9^.

Several studies have emphasized post-pandemic depression as a significant mental health challenge^10–12^. The word “Corona-Blue” has been used to represent depression and lethargy due to self-isolation and social distancing in associated research^13,14^. Moreover, the possible determinants of depression were investigated to identify the factors affected by the pandemic. The prevalent social and economic factors that were established as major factors before the pandemic were found to be identical after the pandemic^15,16^. Furthermore, pandemic-related factors, including fear of job loss and the lockdown, were confirmed through statistical validations^17,18^.

Based on the various factors related to depression, the collected dataset comprised multiple variables in diverse categories. To identify latent relationships within multivariable datasets, statistical modeling or factor analysis methods have been utilized in previous studies^19–21^. For example, statistical modeling, including multivariable linear regression (MLR) models, has been used to interpret the associations of variables with their respective coefficients^22,23^. In addition, exploratory and confirmatory factor analysis methods have been widely applied to examine symptom dimensions (e.g., insomnia and atypical symptoms) in a large community-based cohort^24–26^. These factor analysis methods need to set prior information (e.g., application of the number of factors or hypotheses by related theories)^27,28^.

However, data-driven methods, including machine learning (ML) or deep learning (DL) algorithms without specific prior criteria have been widely used in recent studies. Notably, ML algorithm performances were mainly used as evaluation criteria to validate the impact of diverse factor candidates^29,30^. Additionally, feature importance results, which can be calculated through the working of ML algorithms (e.g., coefficients of support vector machine or f-score from XGBoost algorithms), are utilized to compare the relative effects between variables in datasets^31,32^. Furthermore, several DL algorithms have proposed specialized modules in their process to show important feature sets from input datasets. To detect depression using diverse categories of datasets, self-attention or attention mechanism modules have been applied to analyze the features noticed by algorithms^33,34^.

Unlike the two methods (statistical and data-driven methods) mentioned above, a quantum annealing (QA)-based optimization method has been introduced for feature selection in existing studies^35,36^. These methods entail the optimization of energy state (from the initial state to the lowest energy state) in commercial quantum annealers of D-Wave devices. For the selection of various biosignal features to detect stress level, an automated feature selection framework with quantum annealer was proposed^37^. In addition, optimization algorithms using quantum annealing have been applied to select the optimized transcription factors for DNA sequence^38^.

Following the recent application of quantum annealers for feature selection tasks, we developed a feature selection algorithm with QA-based optimization. This algorithm contains an iterative combinational optimization process to find the optimal features from input feature sets executed by QA-based optimization. To examine the performance of quantum annealers in feature selection tasks, researchers have used diverse methods that have been applied in previous studies. Nath et al. used mutual information and correlation as a feature selection method for comparisons^37^. Similar to these comparisons, we selected the XGBoost algorithm that was widely applied for feature selection in previous studies^39,40^. The details of the two algorithms (QA-based feature selection and XGBoost) are described in the Methods section.

In this study, we investigated the changes in the relative importance of variables before and after the pandemic using a survey dataset collected from college student groups on depression. Among the three aforementioned methods that could be applied for factor investigation, we focused on the QA-based feature selection algorithm to validate the algorithm performance through comparisons with MLR models (statistical method). Moreover, XGBoost models (data-driven method) were utilized to evaluate the selected features from the two algorithms (i.e., QA-based algorithms and statistical analysis methods) using their classification and regression performances as quantitative indices. Finally, we confirm the variables with their rank changes after the pandemic using trends proposed in previous studies as a qualitative evaluation tool. The main contributions of this study are as follows:

- We investigated the changes in important variables for college students’ depression after the pandemic.
- Quantum annealing-based feature selection algorithms were proposed and applied in the analysis.
- The proposed algorithms were validated using real-world survey datasets and classical statistical machine learning models.

## Results

### XGBoost algorithm performance of selected features from two feature selection algorithms

To evaluate the selected feature sets from the D-Wave QA algorithms and MLR models, we checked the performance of the XGBoost algorithms under the classification and regression tasks. Moreover, experiments with five evaluation conditions based on accumulated independent variables (i.e., top 1–10, top 1–20, top 1–30, top 1–40, and top 1–50 variables) were conducted to compare the influence of the selected features on the performance of the XGBoost algorithm. All experimental conditions were repeated 30 times to validate the differences between the conditions using t-test.

We found that the overall performance indices (classification: balanced accuracy; regression: negative mean squared error) of the QA-based algorithms were higher than those of the MLR models. Moreover, the differences in the indices between the two methods (QA-based algorithms and MLR models) in all the experimental results were confirmed to be statistically significant (*p-value* < 0.05). The detailed experimental results are presented in Tables 1 and 2.

**Table 1:** Average classification performance (balanced accuracy) of the XGBoost algorithm.

Experimental conditions	Top 10 (top 1–10)	Top 20 (top 1–20)	Top 30 (top 1–30)	Top 40 (top 1–40)	Top 50 (top 1–50)
Dependent variable	Before the COVID-19 pandemic				
Quantum annealer	0.745	0.743	0.736	0.741	0.746
Multivariable linear regression	0.589	0.604	0.614	0.620	0.621
Dependent variable	After the COVID-19 pandemic				
Quantum annealer	0.750	0.725	0.735	0.743	0.748
Multivariable linear regression	0.580	0.605	0.635	0.629	0.629

The differences in all the averaged performance indices (QA vs. MLR) were statistically significant (*p-*value < 0.05).

**Table 2:** Average regression performance (negative mean absolute error) of the XGBoost algorithm.

Experimental conditions	Top 10 (top 1–10)	Top 20 (top 1–20)	Top 30 (top 1–30)	Top 40 (top 1–40)	Top 50 (top 1 ~ 50)
Dependent variable	Before the COVID-19 pandemic				
Quantum annealer	− 3.152	− 2.950	− 2.870	− 2.819	− 2.829
Multivariable linear regression	− 4.531	− 4.152	− 4.051	− 3.989	− 3.948
Dependent variable	After the COVID-19 pandemic				
Quantum annealer	− 2.959	− 2.959	− 2.819	− 2.820	− 2.798
Multivariable linear regression	− 4.386	− 4.168	− 3.988	− 3.843	− 3.852

The differences in all the averaged performance indices (QA vs. MLR) were statistically significant (*p-*value < 0.05).

### Comparisons of variables with changes of importance in the post-pandemic condition

Based on comparisons of XGBoost algorithm performances, we identify that QA-based feature selection algorithms have comparable feature selection capabilities to MLR models, which have been widely utilized in previous studies. Based on the aforementioned trends, we checked the variables with higher ranks in the post-pandemic conditions calculated using QA-based algorithms. In the top 1 to 10 ranks, the ranks of pandemic- and virus-related variables were higher after the pandemic than before the pandemic. For example, the importance of social interactions with family, friends, and third parties (“q226” and “q143”) and reliability of the public medical system (“q300") became high.

Similarly, in the top 11 to 50, pandemic (or virus)-related variables and decision-making-associated variables showed higher ranks in post-pandemic conditions. Social distancing (“q303”), safety of family members in pandemic (“q299”), infections about contact with foreigners (“q163”), and exaggerations of COVID-19 dangers (“q159”) showed relatively higher importance than before pandemic conditions. In addition, uncertain situations (“q77"), deferred decision-making (“q186”), and deferred decision-making with anxious thoughts (“q134”) were also found to have higher ranks in the post-pandemic conditions. The detailed changes in the variables and survey questions for each confirmed variable are presented in Appendices A and B.

## Methods

### Study design

**Figure 1:**
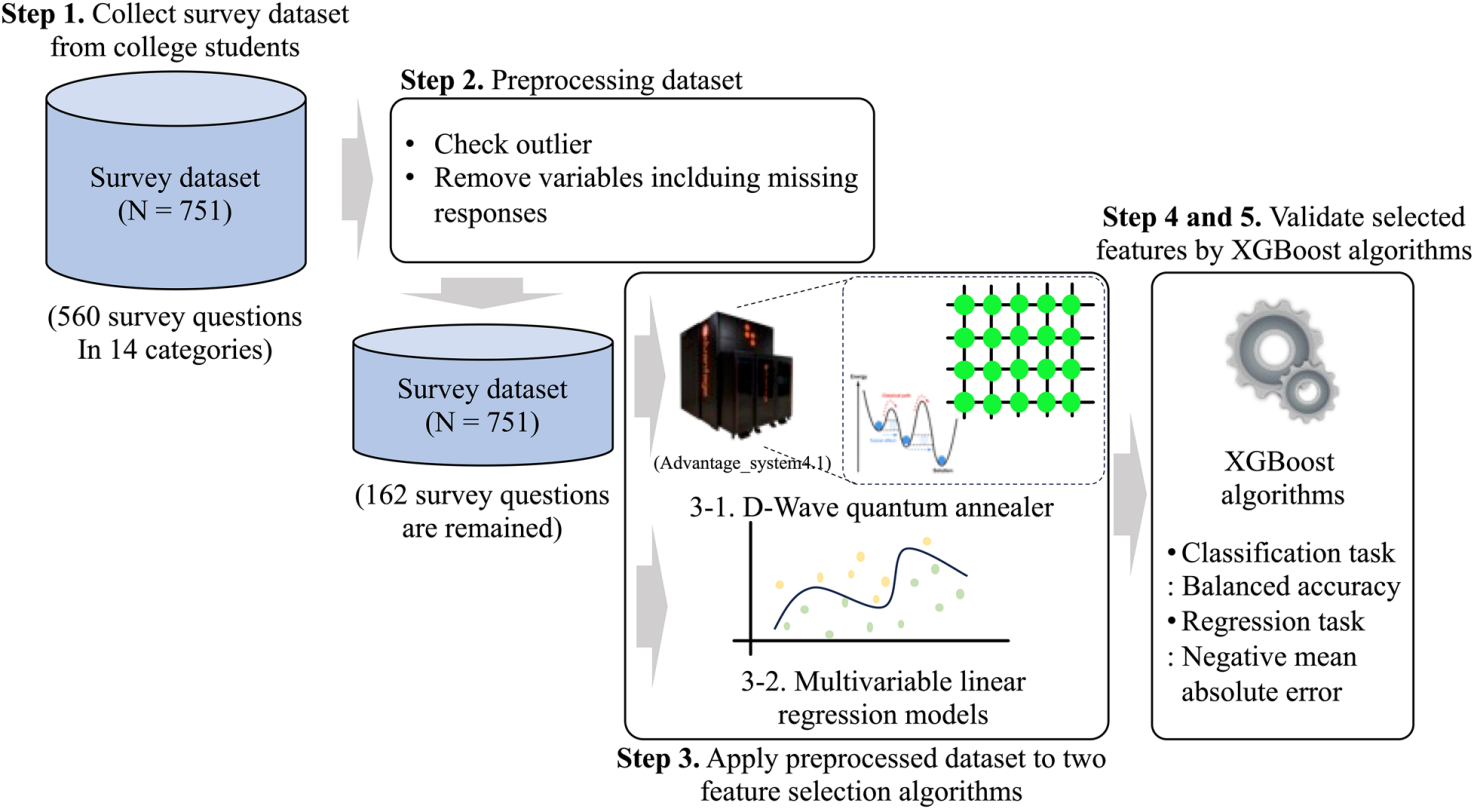
Overview of the study design.

To compare the different factors before and after the COVID-19 pandemic, our study adopted five steps. First, we collected survey datasets from 751 college students using 560 survey questions in 14 categories, including demographics, pre-pandemic, and post-graduation. Second, the collected survey datasets were preprocessed by removing variables with missing values and checking for possible outliers. Third, the preprocessed datasets were applied to quantum annealer and MLR models to determine the relative importance of the variables from each algorithm. Fourth, the variables from the two algorithms (quantum annealer and MLR models) were validated using XGBoost algorithms with regression and classification performance. Finally, the rank change of the variables from the algorithm that exhibited better performance was confirmed. Our overall study design is depicted in Fig. 1.

### Datasets and preprocessing

Survey questionnaires were administered to investigate the mental health status of college students before and after the pandemic^41^. This questionnaire included 560 survey questions in 14 categories (e.g., anxiety, depression, suicide, panic, and traumatic events). Responses were collected from college students using EMBRAIN, an online survey company in Korea. A total of 751 college students participated in the data collection and were rewarded (Male: 220, Female: 531, Age: 22.15±2.15). The dimension of the raw survey dataset was (751, 560) (i.e., 751 rows and 560 columns).

Before applying the algorithms for variable importance, we removed variables (columns) with missing values. After removing columns with missing values, the shape of the dataset changed to (751, 270) (i.e., 290 variables were removed). As the survey variables, except demographics, consisted of only categorical survey questions, outlier removal was not considered in this preprocessing. Among the diverse variables related to mental health, 18 depression-related variables were set as the dependent variables. Because mental health variables constituted variables about “About 2 weeks before the COVID-19 pandemic” (i.e., before pandemic) and variables about “In the past 2 weeks” (after pandemic), nine variables each for before and after the pandemic were summed to single variables. Furthermore, all variables with similar content were manually excluded by researchers based on the survey questions (e.g., two similar survey questions about the usage time change of SNS during the pandemic). The final dataset comprised 751 rows and 162 columns (variables).

### D-Wave quantum processor unit (QPU)

D-Wave QPU is built based on the Ising model from statistical mechanics. This model consists of lattice structures with nodes and edges^42^. In each node, spins are located as a binary variable. In the D-Wave processor, superconducting flux qubits were used to represent the spins. The variables in the Ising model indicate spin-up and spin-down states that correspond to + 1 and − 1 values, respectively. Correlations between spins are calculated to represent their relationships. The objective function of the Ising model is given as follows:

1 $$ E_{i\sin g} \left( S \right) = \sum\limits_{i = 1}^{N} {h_{{_{i} }} } s_{i} + \sum\limits_{i = 1}^{N} {\sum\limits_{j = i + 1}^{N} {J_{i,j} } } s_{i} s_{j} $$

where ${h}_{i}$ represents the bias of the *i*th qubit and ${s}_{i}$ indicates spin in the spin set (${s}_{i}\in S$) of the quantum system. In addition, ${J}_{i,j}$ is the coupling strength between *i*th spin and *j*th spin and ${E}_{ising}(S)$ denotes the Hamiltonian of the quantum system at a given state $S$. An optimization process aims to minimize the energy state to figure out the ground state of the system.

One can leverage the Ising model to solve diverse optimization problems by translating it into a binary quadratic model (BQM)^43,44^. The objective function of the BQM is stated as follows:

2 $$f\left(x\right)=\sum_{i}{Q}_{i,i}{x}_{i}+\sum_{i<j}{Q}_{i,j}{x}_{i}{x}_{j}={x}^{T}Qx,$$

where ${Q}_{i,j}$ is the element in the *i*th row and *j*th column of a real-valued upper-triangular matrix $Q$ and ${x}_{i}\in \{\mathrm{0,1}\}$ is the *i*th element of $x$. The objective function $f(x)$ is minimized to find optimal solutions (i.e., ${argmin}_{x\in {\{\mathrm{0,1}\}}^{n}} {x}^{T}Qx$) for a given coefficient matrix Q.

### Mapping features to D-Wave quantum annealer

We use mutual information (MI) to reflect the correlation between features and conditional information (CMI) to incorporate relationships between features and dependent variable. CMI values should be negated in the minimization process since we want to keep CMI maximized. The MI and CMI are defined as follows:

3 $$I\left(X;Y\right)=H\left(X\right)-H\left(X|Y\right),$$

4 $$I\left(X;Y|Z\right)=H\left(X|Z\right)-H\left(X|Y,Z\right),$$

where $I(X;Y)$ and $I(X;Y|Z)$ represent MI and CMI, respectively. In formulas (3) and (4), $H(X)$ denotes the marginal entropy of $X$ quantifying the amount of information contained in features and $H(X|Y)$ indicates the conditional one. We put negative of CMI’s as diagonal terms and MI’s as upper-triangular terms of coefficient matrix $Q$.

The BQM library included in D-wave Ocean software converts the objective function to a graph, where $V$ denotes a set of vertices and $E$ denotes a set of edges connecting vertices that encodes MI’s and CMI’s and embeds it to D-Wave’s QPU. To select the best feature combination, BQM goes through iterative optimization process for a given number of features $k$, that goes from 1 to 161. After the entire process, we get the relative importance of features by counting the number of appearances. For those who want to find more details about graph level implementations, see Ref.^45^.

### Statistical models for comparison

To compare the feature selection results from the D-Wave QA based algorithms, we used MLR models. The same features and dependent variables were used in the MLR models. The coefficient values of each feature from the fitted MLR models were set as the criteria for relative associations with the dependent variables.

### XGBoost algorithms for the validation of selected feature sets

After organizing the two selected feature sets calculated from the two algorithms (D-Wave QA-based algorithms and MLR models), we need to validate the feature selection results. Among the various applicable methods, XGBoost algorithms, which were widely known in classical ML algorithms, were selected as an evaluation model^46,47^. The classification and regression performance index values from the trained XGBoost algorithms were compared for the two feature selection algorithms. The dependent variables were additionally converted from continuous to binary categorical variables for evaluation in the classification task of the XGBoost algorithms. Among the 162 selected variables with their ranks, we conducted accumulative evaluations with 5 variable conditions based on the rank of each variable (top 10, top 20, top 30, top 40, and top 50). Balanced accuracy and negative mean absolute error were utilized as performance indices for each classification and regression task. A tenfold cross validation was also used to evaluate the performance of the XGBoost algorithms under strict conditions. The same procedure was repeated 30 times to validate the classification performance between the experimental conditions based on independent two-sample t-tests.

*Tools.* D-Wave QA-based feature selection algorithm codes were written using the D-Wave Ocean software (Python-based software). Data preprocessing, MLR models, and XGBoost classifiers were built and operationalized using Python (version 3.7.1; scikit-learn, version 2.4.1) and R (version 4.0.3).

### Ethics approval

This study was approved by the Institutional Review Board (IRB) Committee of Yonsei University (BO-EK-156042020), and all tests were carried out in accordance with relevant guidelines and regulations.

### Consent to participate

Informed consent was obtained from all individual participants included in the study.

## Discussion

In this study, we investigated the factors affecting depression in college student groups by comparing depression-related factors during the pre-pandemic period. To compare the appropriacy of diverse feature selection algorithms, we considered three types of algorithms in our study design (i.e., statistical modeling, data-driven modeling, and QA-based algorithms). Among the three algorithms considered, we focused on appraising the feature selection capability of the QA-based algorithms through comparisons with statistical modeling (MLR models). Feature sets with importance ranks calculated from the QA-based algorithms and MLR models were validated using data-driven methods (XGBoost algorithms under classification and regression tasks) to evaluate each feature selection algorithm.

Various feature selection methodologies have been utilized to identify the major factors in the datasets in previous studies^48,49^. To examine the applicability of QA-based algorithms for feature selection tasks, we applied MLR models, which could be considered as typical methods utilized in associated studies^50^. In addition, XGBoost algorithms are widely used in ML models for multivariate analysis as validation models to evaluate selected features based on their importance, according to algorithm performances^51^. Two performance indices (balanced accuracy and negative mean-squared error) were used for the classification and regression tasks.

For validation experiments with XGBoost algorithms, we created experimental conditions with accumulated variables based on the rank of each variable. Five conditions were fixed with groups including 10 variables (i.e., condition 1: top 1–10, condition 2: top 1–20, condition 3: top 1–30, condition 4: top 1–40, and condition 5: top 1–50). We hypothesize that if we could check a similar scale of performance indices in the overall conditions, it indicates that the 50 features selected from each algorithm have sufficient associations with depression in college student groups (dependent variables). Moreover, if higher performance indices are established in QA-based algorithms, it indicates that such QA-based algorithms can show capabilities similar to those of existing methods that have been applied in previous studies.

In the classification tasks with selected features, we verified that the QA-based algorithms showed higher performance (higher balanced accuracy) in all experimental conditions. Similarly, in the regression tasks, smaller negative mean absolute errors were ascertained using the QA-based algorithms in all experimental conditions. From the aforementioned results, we confirmed that the QA-based feature selection algorithm is competitive compared with the MLR models. Furthermore, performance index values showed similar trends from condition 1 (top 1–10 variables) to condition 5 (top 1–top 50), and these were found in most experimental conditions except the “after pandemic” condition in classification tasks. Balanced accuracy in the classification tasks increased and negative mean absolute error in the regression tasks decreased from condition 1 to condition 5 (i.e., balanced accuracy in condition 5 was higher than that in condition 1, and negative mean absolute error in condition 5 was lower than that in condition 1). Based on these trends, we also checked the feature selection capacity of the QA-based algorithms.

Based on the quantitative evaluation mentioned above, we confirm that the QA-based feature selection algorithms are applicable to feature selection tasks based on comparisons with the methods utilized in previous studies. Based on these results, we compared the important features before and after the pandemic using QA-based algorithms.

Among the diverse variables that needed to be validated, COVID-19-related variables showed more significant changes with higher ranks after pandemic than before it. Especially, variables about social interactions in pandemic were checked in the top-10 category (e.g., “q226”: “Who has helped you during the COVID-19 pandemic period?” and “q143”: “Since the COVID-19 pandemic began, how many meetings have you attended with family, friends, or third parties?”). Similar trends regarding the impact of social interaction during pandemics on depression have been reported in previous studies^52,53^. In addition to the depression of older adult groups during the pandemic, research on the influence of depression on young adults and adolescent groups during the pandemic was conducted^54,55^. Constraints such as the COVID-19 home confinement and school concerns were found to be the main factors vis-à-vis depression among young adults and adolescents^56^. Moreover, the importance of social relationship replacements (e.g., social media usage) was checked for depression and loneliness in the young adult group^57^.

Additionally, social systems, including public medical system-related variables, showed increased ranks after the pandemic. Variables about the concern about the public medical system and safety of family were checked (e.g., “q300”: “I'm afraid the healthcare system won't protect my loved ones” and “q299”: “I'm worried that I won't be able to keep my family safe from the virus”). Furthermore, social-constraint-related variables also showed higher ranks in after pandemic (“q303”: “I worry that social distancing won't be enough to keep me safe from the virus” and “q157”: “The government should open the area I live in without shutting it down due to COVID-19”). Concerns about social or medical systems have been investigated as factors related to depression in young adults in associated studies^18,58^. Relationships between social distancing, including quarantine, with psychosocial consequences, and well-being have been confirmed in young adult groups^59^. Unlike external elements in a pandemic (e.g., social distancing or social systems), internal elements, including psychological factors, were examined together with higher ranks in our experimental results. For example, hope about the future and leaving from uncertain situations were found (“q331”: “I am always hopeful about my future” and “q77”: “I have to get away from all uncertain situations”).

In summary, we verified that QA-based algorithms can be used for feature selection in multivariable datasets, based on comparisons with MLR models (quantitative evaluations). Moreover, the selected variables related to depression in college student groups (i.e., young adult groups) by QA-based algorithms included several variables in both the external and internal element categories (qualitative evaluations). These trends in the selected variables were validated with previous studies.

## Conclusion

In this study, we analyzed the important variables related to depression in college student groups before and after the COVID-19 pandemic using QA-based feature selection algorithms. The QA-based algorithms, executed on the D-Wave QPU, are validated by comparisons with MLR and XGBoost algorithms, which have been widely utilized in previous studies. Through validation experiments, we identify that QA-based algorithms have a feature selection capability that is as good as that of previously applied methods. Social interactions and social systems in the pandemic-related variables ranked higher after the pandemic. Moreover, psychological factor variables, including decision-making in uncertain situations and hope for the future, ranked higher after the pandemic. These results were additionally verified with previous studies.

Our study has several strengths from diverse perspectives. First, we investigated the important variables of depression levels among college students after the COVID-19 pandemic. Second, we proposed a quantum annealing approach feature selection algorithm using D-Wave hardware to check variable importance in real-world survey datasets collected for psychological research purposes. Third, the QA-based feature selection algorithms are compared with classical statistical machine learning models for algorithm validation. However, our study has some limitations. First, to generalize our research conclusions, an analysis of datasets collected from other countries should be conducted using the same research scheme. Second, we need to analyze datasets for other mental-health-related variables (e.g., anxiety or loneliness) to identify complex variables for the overall mental health status of college students.

## Data Availability

The survey datasets analyzed in this study were obtained via EMBRAIN (survey company in Korea). The dataset is not available for public access due to our discretion. For inquiries regarding the data or for further information, please contact the authors directly.

## Appendix A

Changes in variable ranks before and after the COVID-19 pandemic from QA-based feature selection algorithms (top 1 ~ top 50).

**Table:**

Rank	Before	After	Rank	Before	After	Rank	Before	After	Rank	Before	After	Rank	Before	After
1	q_319	q_319	11	q331	q302	21	q77	**q186**	31	q73	q260	41	q298	q262
2	q2_1	q2_1	12	q330	**q264**	22	q265	q265	32	q159	q148	42	q157	q164
3	q317	q317	13	q266	q266	23	q186	q149	33	q255	q71	43	q329	**q174**
4	q318	q318	14	q143	q330	24	q258	**q259**	34	q163	q165	44	q267	**q161**
5	q263	**q226**	15	q327	q332	25	q262	**q261**	35	q261	**q157**	45	q173	**q181**
6	q226	**q143**	16	q148	q301	26	q71	**q163**	36	q162	**q267**	46	q174	q75
7	q302	**q327**	17	q264	**q303**	27	q165	q258	37	q181	**q72**	47	q168	q76
8	q332	**q300**	18	q300	q328	28	q260	**q255**	38	q252	q251	48	q161	q179
9	q328	**q331**	19	q149	**q77**	29	q259	**q184**	39	q75	q73	49	q250	q298
10	q301	q263	20	q303	**q299**	30	q184	**q159**	40	q299	q162	50	q72	q256

Variables with higher rankings in after pandemic conditions are highlighted in bold.

## Appendix B

Survey questions of highlighted variables with higher ranks in the post-COVID-19 pandemic condition.

**Table:**

No	Rank of variables	Survey question code	Survey question
1	5	q226	Who has helped you during the COVID-19 pandemic period?
2	6	q143	Since the COVID-19 pandemic began, how many meetings have you attended with family, friends, or third parties?
3	7	q327	I can imagine what my life will be like in 10 years
4	8	q300	I'm afraid the health care system won't protect my loved ones
5	9	q331	I am always hopeful about my future
6	12	q264	I prefer work that is important and intellectually challenging to work that is important but does not require much thought
7	17	q303	I worry that social distancing won't be enough to keep me safe from the virus
8	19	q77	I have to get away from all uncertain situations
9	20	q299	I'm worried that I won't be able to keep my family safe from the virus
10	21	q186	I defer decision-making whenever possible
11	24	q259	I find it fascinating to rely on thinking to reach the top
12	25	q261	Learning new ways of thinking doesn’t excite me
13	26	q163	I am concerned about contact with foreigners as foreigners may be infected with the virus
14	28	q255	I like to contemplate for a long time
15	29	q184	The thought of having to make a decision makes me anxious, so I keep putting it off
16	30	q159	The dangers of COVID-19 have been greatly exaggerated
17	35	q157	The government should open the area I live in without shutting it down due to COVID-19
18	36	q267	I often find myself thinking about matters that do not affect me personally
19	37	q72	I have to be able to plan everything in advance
20	43	q174	I tend to rely on intuition when making decisions
21	44	q161	I am concerned that foreigners are spreading the virus in our country
22	45	q181	I often make decisions impulsively

## Code availability

The code of applied algorithms in this study is available at https://github.com/Jungguchoi/quantum_annealing_based_feature_selection.
